# Supplementary material for: Photonic terahertz phased array via selective excitation of nonlinear Pancharatnam-Berry elements
Source: Nat Commun. 2025 Sep 1;16:8159. doi: 10.1038/s41467-025-63127-5 (PMC12402293; doi:10.1038/s41467-025-63127-5)
Supplement: Supplementary file 1 — Supplementary Information [file 41467_2025_63127_MOESM1_ESM.pdf]

Supplementary Information for

Photonic terahertz phased array via selective excitation of nonlinear Pancharatnam-Berry elements

Li Niu[1,#], Xi Feng[1,#], Xueqian Zhang[1,*], Yongchang Lu[1], Qingwei Wang[1], Quan Xu[1], Xieyu Chen[1], Jiajun Ma[1], Haidi Qiu[1], Wei E.I. Sha[2], Shuang Zhang[3], Andrea Alù[4,5,*], Weili Zhang[6,*], Jiaguang Han[1,7,*]

[1]Center for Terahertz Waves, State Key Laboratory of Precision Measurement Technology and Instruments, Tianjin University, Tianjin 300072, China
[2]Key Laboratory of Micro-nano Electronic Devices and Smart Systems of Zhejiang Province, College of Information Science & Electronic Engineering, Zhejiang University, Hangzhou 310027, China;
[3]Department of Electrical & Electronic Engineering, University of Hong Kong; 999077, Hong Kong, China
[4]Photonics Initiative, Advanced Science Research Center, City University of New York, New York, 10031, USA.
[5]Physics Program, Graduate Center, City University of New York, New York, 10016, USA.
[6]School of Electrical and Computer Engineering, Oklahoma State University, Stillwater, Oklahoma 74078, USA
[7]Guangxi Key Laboratory of Optoelectronic Information Processing, School of Optoelectronic Engineering, Guilin University of Electronic Technology, Guilin 541004, China
[#]These authors contributed equally to this work
[*]Corresponding authors. Email: alearn1988@tju.edu.cn, aalu@gc.cuny.edu, weili.zhang@okstate.edu, jiaghan@tju.edu.cn

Note 1. Derivation of nonlinear PB phase for THz emission

For a second-order nonlinear difference frequency generation (DFG) process under normal pump, the corresponding effective second-order nonlinear polarization can be expressed as [S1]:

$$\begin{bmatrix} P_x(\omega_{\mathrm{THz}}) \\ P_y(\omega_{\mathrm{THz}}) \end{bmatrix} = \varepsilon_0 \begin{bmatrix} \chi^{(2)}_{xxx} & \chi^{(2)}_{xxy} & \chi^{(2)}_{xyx} & \chi^{(2)}_{xyy} \\ \chi^{(2)}_{yxx} & \chi^{(2)}_{yxy} & \chi^{(2)}_{yyx} & \chi^{(2)}_{yyy} \end{bmatrix} \begin{bmatrix} E_{1x}(\omega_1)E^*_{2x}(\omega_2) \\ E_{1x}(\omega_1)E^*_{2y}(\omega_2) \\ E_{1y}(\omega_1)E^*_{2x}(\omega_2) \\ E_{1y}(\omega_1)E^*_{2y}(\omega_2) \end{bmatrix}. \tag{S1}$$

where the subscripts *x*, *y* of nonlinear THz polarization *P* and pump laser field *E* represent their corresponding polarization components, the subscripts of nonlinear susceptivity *ijk* represent the generation process of *i*-polarized nonlinear THz polarization from *j*-polarized pump laser field E_1 and *k*-polarized pump laser field E_2 with $i, j, k \in \{x, y\}$. Due to the mirror symmetry of the SRR (suppose the mirror plane is along the *y* direction), the second-order susceptibility tensor can be reduced by $\chi^{(2)}_{xxx} = \chi^{(2)}_{yxy} = \chi^{(2)}_{yyx} = \chi^{(2)}_{xyy} = 0$. Then, Eq. (S1) can be calculated as:

$$\begin{bmatrix} P_x(\omega_{\mathrm{THz}}) \\ P_y(\omega_{\mathrm{THz}}) \end{bmatrix} = \varepsilon_0 \begin{bmatrix} 0 & \chi^{(2)}_{xxy} & \chi^{(2)}_{xyx} & 0 \\ \chi^{(2)}_{yxx} & 0 & 0 & \chi^{(2)}_{yyy} \end{bmatrix} \begin{bmatrix} E_{1x}(\omega_1)E^*_{2x}(\omega_2) \\ E_{1x}(\omega_1)E^*_{2y}(\omega_2) \\ E_{1y}(\omega_1)E^*_{2x}(\omega_2) \\ E_{1y}(\omega_1)E^*_{2y}(\omega_2) \end{bmatrix}. \tag{S2}$$

Consider our case that the SRR is rotated by *θ*, see Fig. S1, Eq. (S2) is still satisfied in its local coordinate *x'y'z'*, i.e.,

$$\begin{bmatrix} P_{x'}(\omega_{\mathrm{THz}}) \\ P_{y'}(\omega_{\mathrm{THz}}) \end{bmatrix} = \varepsilon_0 \begin{bmatrix} 0 & \chi^{(2)}_{x'x'y'} & \chi^{(2)}_{x'y'x'} & 0 \\ \chi^{(2)}_{y'x'x'} & 0 & 0 & \chi^{(2)}_{y'y'y'} \end{bmatrix} \begin{bmatrix} E_{1x'}(\omega_1)E^*_{2x'}(\omega_2) \\ E_{1x'}(\omega_1)E^*_{2y'}(\omega_2) \\ E_{1y'}(\omega_1)E^*_{2x'}(\omega_2) \\ E_{1y'}(\omega_1)E^*_{2y'}(\omega_2) \end{bmatrix}. \tag{S3}$$

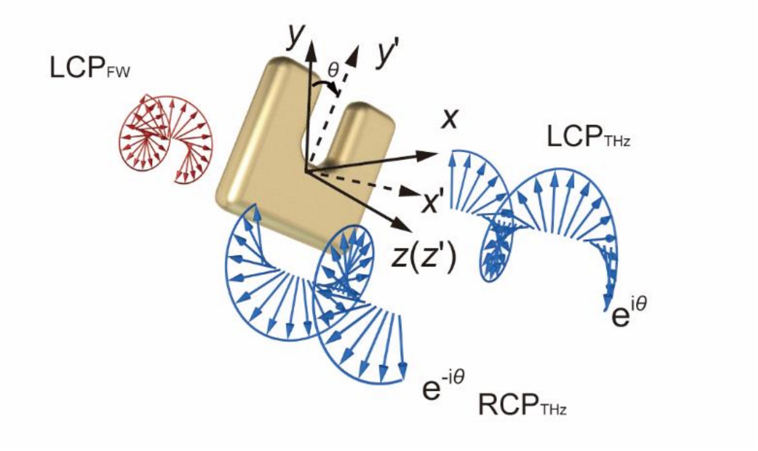

Fig. S1. Schematic of the nonlinear Pancharatnam–Berry (PB) phases for circularly polarized terahertz (THz) generations under circularly polarized pump.

Suppose the pump laser field in the global coordinate *xyz* is circularly polarized with $E_x = 1/2^{1/2}$ and $E_y = \sigma i/2^{1/2}$, where $\sigma = +1$ and $\sigma = -1$ represent LCP and RCP, respectively. Then, the corresponding pump laser field in the local coordinate can be calculated as

$$\begin{bmatrix} E_{x'} \\ E_{y'} \end{bmatrix} = R \begin{bmatrix} E_x \\ E_y \end{bmatrix} = \frac{1}{\sqrt{2}} \begin{bmatrix} \cos\theta & \sin\theta \\ -\sin\theta & \cos\theta \end{bmatrix} \begin{bmatrix} 1 \\ \sigma i \end{bmatrix} = \frac{e^{i\sigma\theta}}{\sqrt{2}} \begin{bmatrix} 1 \\ \sigma i \end{bmatrix}, \tag{S4}$$

with *R* being rotation matrix. Substituting Eq. (S4) into Eq. (S3) gives,

$$\begin{bmatrix} P_{x'}(\omega_{\mathrm{THz}}) \\ P_{y'}(\omega_{\mathrm{THz}}) \end{bmatrix} = \frac{1}{2}\varepsilon_0 \begin{bmatrix} \sigma i \left(\chi^{(2)}_{x'y'x'} - \chi^{(2)}_{x'x'y'} \right) \\ \chi^{(2)}_{y'x'x'} + \chi^{(2)}_{y'y'y'} \end{bmatrix}. \tag{S5}$$

Transforming Eq. (S5) back to the global coordinate gives,

$$\begin{aligned} \begin{bmatrix} P_x(\omega_{\mathrm{THz}}) \\ P_y(\omega_{\mathrm{THz}}) \end{bmatrix} &= R^{-1} \begin{bmatrix} P_{x'}(\omega_{\mathrm{THz}}) \\ P_{y'}(\omega_{\mathrm{THz}}) \end{bmatrix} = \frac{1}{2}\varepsilon_0 \begin{bmatrix} \cos\theta & -\sin\theta \\ \sin\theta & \cos\theta \end{bmatrix} \begin{bmatrix} \sigma i \left(\chi^{(2)}_{x'y'x'} - \chi^{(2)}_{x'x'y'} \right) \\ \chi^{(2)}_{y'x'x'} + \chi^{(2)}_{y'y'y'} \end{bmatrix} \\ &= \frac{1}{2}\varepsilon_0 \begin{bmatrix} \sigma i \left(\chi^{(2)}_{x'y'x'} - \chi^{(2)}_{x'x'y'} \right)\cos\theta - \left(\chi^{(2)}_{y'x'x'} + \chi^{(2)}_{y'y'y'} \right)\sin\theta \\ \sigma i \left(\chi^{(2)}_{x'y'x'} - \chi^{(2)}_{x'x'y'} \right)\sin\theta + \left(\chi^{(2)}_{y'x'x'} + \chi^{(2)}_{y'y'y'} \right)\cos\theta \end{bmatrix} \end{aligned}. \tag{S6}$$

Converting Eq. (S6) into circular polarization basis gives,

$$
\begin{aligned}
\begin{bmatrix} P_l(\omega_{\mathrm{THz}}) \\ P_r(\omega_{\mathrm{THz}}) \end{bmatrix} &= C\begin{bmatrix} P_x(\omega_{\mathrm{THz}}) \\ P_y(\omega_{\mathrm{THz}}) \end{bmatrix} \\
&= \frac{1}{\sqrt{2}}\begin{bmatrix} 1 & i \\ 1 & -i \end{bmatrix} * \frac{1}{2}\varepsilon_0 \begin{bmatrix} \sigma i\left(\chi^{(2)}_{x'y'x'} - \chi^{(2)}_{x'x'y'}\right)\cos\theta - \left(\chi^{(2)}_{y'x'x'} + \chi^{(2)}_{y'y'y'}\right)\sin\theta \\ \sigma i\left(\chi^{(2)}_{x'y'x'} - \chi^{(2)}_{x'x'y'}\right)\sin\theta + \left(\chi^{(2)}_{y'x'x'} + \chi^{(2)}_{y'y'y'}\right)\cos\theta \end{bmatrix}, \quad \text{(S7)} \\
&= \frac{1}{2\sqrt{2}}\varepsilon_0 \begin{bmatrix} \left[\sigma i\left(\chi^{(2)}_{x'y'x'} - \chi^{(2)}_{x'x'y'}\right) + i\left(\chi^{(2)}_{y'x'x'} + \chi^{(2)}_{y'y'y'}\right)\right]e^{i\theta} \\ \left[\sigma i\left(\chi^{(2)}_{x'y'x'} - \chi^{(2)}_{x'x'y'}\right) - i\left(\chi^{(2)}_{y'x'x'} + \chi^{(2)}_{y'y'y'}\right)\right]e^{-i\theta} \end{bmatrix}
\end{aligned}
$$

where the subscripts *l* and *r* represent LCP and RCP, *C* represents the transforming matrix from linearly polarized basis to circularly polarized basis. Clearly, nonlinear PB phases of θ for LCP terahertz component and $-\theta$ for RCP terahertz component are observed.

In general, $\chi^{(2)}{}_{yyy} = 0$ and $\chi^{(2)}{}_{yxx}$ is much superior than $\chi^{(2)}{}_{xyx}$ and $\chi^{(2)}{}_{xxy}$ in SRR[S2,S3], thus Eq. (S7) can be further simplified to,

$$
\begin{bmatrix} P_l(\omega_{\mathrm{THz}}) \\ P_r(\omega_{\mathrm{THz}}) \end{bmatrix} = \frac{i\varepsilon_0\chi^{(2)}_{y'x'x'}}{2\sqrt{2}}\begin{bmatrix} e^{i\theta} \\ e^{-i\theta} \end{bmatrix}. \quad \text{(S8)}
$$

In this case, the LCP and RCP terahertz waves have nearly the same amplitude.

More importantly, the above derivation does not involve any dispersion effect, indicating that the nonlinear PB phase is naturally dispersionless, forming the foundation of the broadband feature of our PTPA.

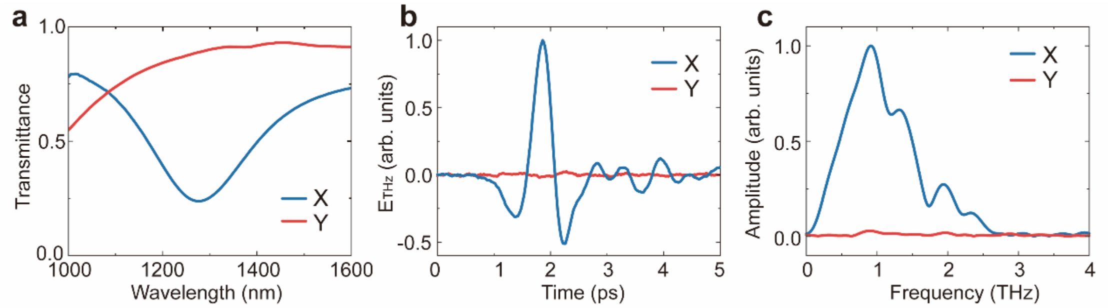

Fig. S2 Transmittance and terahertz (THz) generation responses of the uniform split-ring resonator (SRR) array. a Measured transmittance spectra of the uniform SRR array under the *x*- and *y*-polarized incidences. **b,c** Measured normalized time-domain THz pulses and corresponding frequency-domain spectra under the *x*- and *y*-polarized pumps at 1275 nm. Characterizing the property of sub-elements poses challenges due to their varied orientations. Therefore, the above data is measured by a uniform array with a size of 500 μm × 500 μm.

Note 2. Detour phase of the sub-element

In our design, the four types of sub-elements are all not uniformly arranged within each element, this would cause additional non-uniform propagation phase differences for oblique THz radiation, which are also known as the "detour phase"[S4]. For an arbitrary 2D deflection angle (θ_x, θ_y), the detour phase can be calculated as $\varphi_d = k(x\sin\theta_x + y\sin\theta_y)$ with x and y being the location of the sub-element.

1) For the case of normal and small-angle THz radiation, the detour phase is small. The phase of each sub-element is sole determined by the nonlinear PB phase. Thus, when the sub-elements of the same orientation being excited, their nonlinear PB phase can roughly be considered as the phase of each element, see Fig. 2d in the main text.

2) For the case of beam steering along the x or the y directions, where $\theta_y = 0$ or $\theta_x = 0$, the THz radiations from the sub-elements of the same orientation in each element can both be expressed as $\exp(i\varphi_{PB})[1 + \exp(i\varphi_d) + \exp(i2\varphi_d) + \exp(i3\varphi_d)]$ with $\varphi_d = kP_2\sin\theta_x$ or $kP_2\sin\theta_y$, respectively, according to the arrangement style of the sub-elements in Fig. 2 (the detour phases are referenced to the one at the element edge). It is seen that the detour phase contributions are the same, the nonlinear PB phase can still be considered as the phase of each element.

3) Whereas for the case of the other deflection angles, the detour phases will affect, whose contribution is determined by the actual angle and will make the final radiation phase fluctuates around the nonlinear PB phase.

In Fig. 2, only nonlinear PB phase is considered to show our phase control scheme in a clear physical picture, since it is the main source of the phase control here.

Note 3. Experimental measurements

All the measurements on PTPA are carried out using a home-made DMD-integrate THz time-domain spectroscopy (TDS) system, as shown in Fig. S3. The setup employs a Ti:sapphire femtosecond laser amplifier (Legend Elite Duo, Coherent, Inc.) as the source, emitting pulses of 800-nm central wavelength, 1.0-kHz repetition rate and 35-fs pulse duration. A beam splitter divides the laser output into two paths: the reflected beam serves as the probe beam of the TDS, while the transmitted beam drives an optical parametric amplifier (OPA, Coherent OperA Solo) to produce a near-infrared (NIR) femtosecond laser beam, which is tunable from 1160 nm to 2000 nm with an approximately 60 fs pulse duration and vertical polarization. It serves as the pump beam of the TDS.

For the probe beam, it first passes through a beam expander composed of two lenses (L4 and L5). After guided by several mirrors, it is reflected by a 2 mm-thick high-resistance silicon wafer (SW) to a 1 mm-thick 110-cut ZnTe crystal. For the pump beam, after passing through a delay line (DL), a chopper and several mirrors, it is encoded by a specially designed DMD-integrated optical path. As DMD functions as a reflective blazed grating, its dispersion to broadband femtosecond laser causes a wavefront tilt in the diffraction beam, resulting in temporal coherence loss and decreasing THz generation efficiency. To mitigate this, a self-compensate method based on a reflection-type $4f$ system is proposed, which is composed of L1 and M with M normally placing at the back focal plane of L1 with focal length $f_1 = 50$ mm. The applied DMD here is DLP4500NIR (Texas Instruments), whose working area is 9.855 × 6.1614 mm^2 consisting of 912 × 1140 micromirrors. Initially, the incident laser beam from the OPA is directed to the left-half area of the DMD in normal incidence with a fluence of 424.4 μJ cm^{-2}, where the micromirrors are all in on-state. Subsequently, the diffraction order with the highest intensity towards –24° is collected and reflected back by the 4-f system in an off-axis manner. Then, the reflected beam with a fluence of 144.7 μJ cm^{-2} is incident onto the right-half area of the DMD with the coding pattern. In this way, the dispersion of the final diffraction beam will be cancelled and acquire a desired beam

pattern at the same time. Finally, this patterned beam is collected and imaged by a 1:1 imaging system comprising L2 (f_2 = 200 mm) and L3 (f_3 = 200 mm) onto the PTPA. The LP and QWP1 placed before the PTPA are used to control the pump polarization.

Next, the emitted THz wave from the PTPA is collected and focused onto a ZnTe crystal by two parabolic mirrors, PM1 and PM2, forming a 4-f imaging system with a magnification of 2. Here, the SW actually function as a reflector for probe beam and a transmitter for THz waves, after which they are adjust collinearly. Inside the ZnTe crystal, the instant THz field modulate the polarization state of the probe beam through electro-optic effect. This probe beam passes through an aperture (APE), a quarter-wave plate (QWP2), and a Wollaston prism (WP) in sequence and finally illuminates onto the balance detector (BD). The signal output from the BD is proportional to the instant THz field. By gradually changing the DL position to alter the encounter time between, the entire time-domain THz pulse can be mapped. The corresponding frequency-domain result can be obtained by taking Fourier transform to the measured pulse.

In the above configuration, the THz spatial distribution at the front focal plane of PM1 is projected onto the back focal plane of PM2, and then detected by the ZnTe crystal positioned in the back focal plane of PM2. However, due to the APE limitations, whose diameter is 0.3 mm, only the THz signal at the back focal point of PM2 is ultimately detected. This implies that only the THz signal at the front focal point of PM1 is detected. Two THz linear polarizers, TLP1 and TLP2, are positioned between PM1 and PM2, where TLP2 is fixed to transmit only the horizontally polarized THz waves while TLP1 is rotated to allow transmission of either −45° or 45° polarized THz waves. This enables us to characterize the polarization state of the generated THz waves.

When measuring the single and dual beamforming functionalities, a 5-mm slit driven by a motorized translation stage is inserted between PM1 and TLP1. Incremental adjustments to the slit position along z direction allow for the detection of the emission angle of the THz beam through a simple geometrical conversion (see Supplementary Note 4). The measured results are then transformed into the circular polarization basis for analysis.

When measuring the imaging functionalities, the same slit between PM1 and TLP1 is applied, but its position is fixed during the entire imaging process. By measuring the THz signals corresponding to each pattern in Tab. S1 in circular polarization basis, the slit can be imaged by synthetizing all the results.

When measuring the vortex beam generation, the ZnTe and APE are mounted on a two-dimensional motorized translation stage, allowing the two-dimensional THz spatial electric field distribution to be scanned point by point. The measured results are then transformed into the circular polarization basis for further analysis.

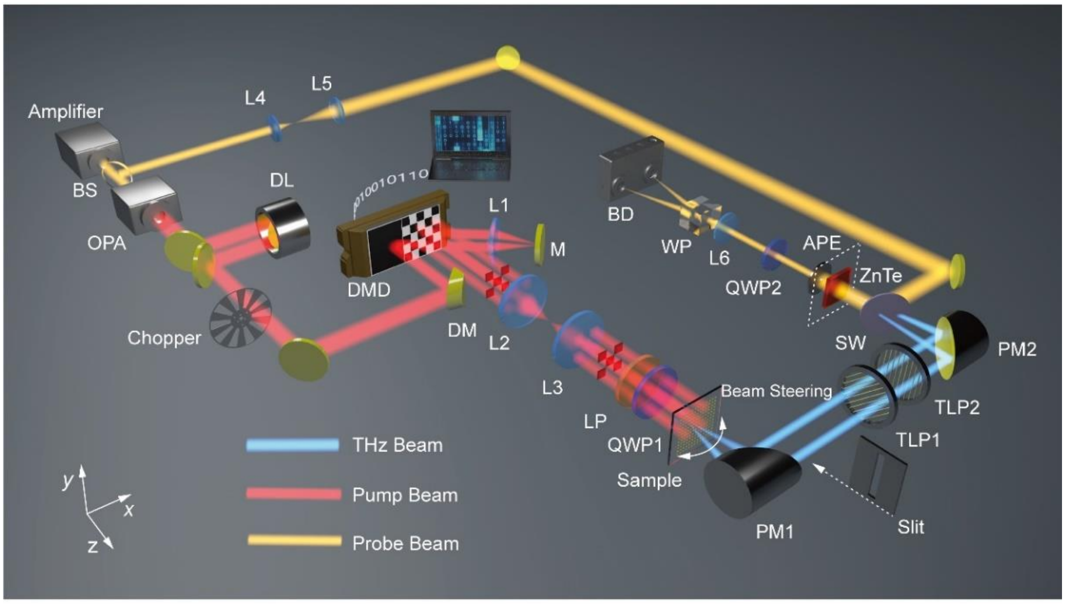

Fig. S3 Experimental setup. Amplifier: femtosecond laser amplifier; BS: beam splitter; OPA: optical parameter amplifier; DL: delay line; DM: D-shaped mirror; L: lens; M: mirror; LP: linear polarizer; QWP: quarter-wave plate; PM: parabolic mirror; TLP: THz liner polarizer; SW: silicon wafer; APE: aperture; WP: Wollaston prism; BD: balanced detector.

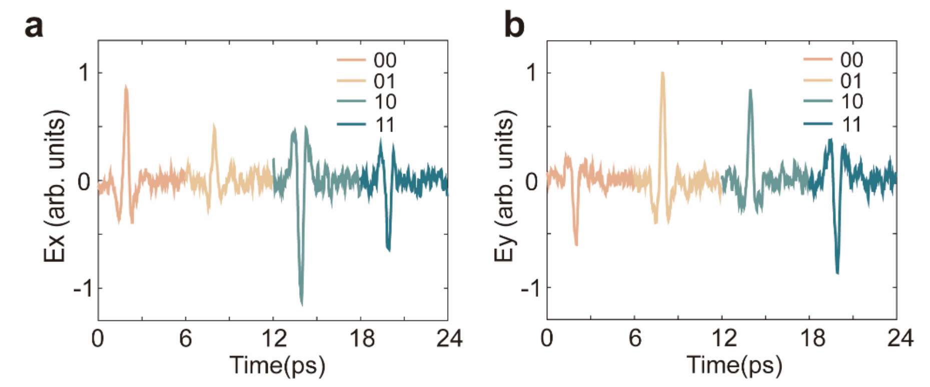

Fig. S4 Measured time-domain terahertz (THz) pulses at different phase states. a,b Measured E_x and E_y components of the time-domain THz signals under different Digital Micromirror Device (DMD) coding patterns in Fig. 2d.

Note 4. Experimental details for the angle-resolved THz intensity distributions

The core section for measuring the angle-resolved THz intensity consists of two parabolic mirrors (PM1, $f = 25.4$ mm, and PM2, $f = 50.8$ mm Thorlabs), a 5-mm-wide metallic slit, and two THz linear polarizers (TLP1 and TLP2), see Fig. S5. The PTPA is placed just at the focal point of PM1. When the PTPA performs beamforming functionality, the emitted THz waves are directed towards specific angles α determined by the phase gradients, which are subsequently collected and collimated by PM1. It is clear that different emission angle α corresponds to different collimated beam position z after PM1. Nonetheless, after collecting by PM2, they are all focused at the focal point of PM2 for detection. According to the parabolic nature of the reflection surface, one can obtain a relation between the emission angle α and the beam position z,

$$\alpha = \begin{cases} \tan^{-1}(z \cdot \tan(\beta_1) / 25.4), z < 0 \\ 0 \qquad\qquad\qquad\qquad , z = 0 \\ \tan^{-1}(z \cdot \tan(\beta_2) / 25.4), z > 0 \end{cases} \tag{S9}$$

Here, $\beta_1 = 36.9°$ and $\beta_2 = 22.6°$ correspond to the collection angle limits on the two sides of the parabolic mirror, respectively. By scanning the position of the slit between PM1 and PM2 along the z direction and recording the transmitted THz time-domain signals at each position, the angle-resolved THz information can be determined. Since the THz beam being controlled here is circularly polarized, two THz linear polarizers (TLP1 and TLP2) before PM2 are used to analyze the polarization information at each slit position. During the measurement, TLP2 is fixed to transmit only horizontally polarized THz waves, while TLP1 is rotated by either –45° or 45° to measure orthogonally polarized field information, i.e., $E_{45°}$ and $E_{-45°}$. Then, the LCP and RCP THz field components E_l and E_r can be extracted using the transforming matrix C in Eq. (S7), as well as the corresponding intensities $|E_l|^2$ and $|E_r|^2$.

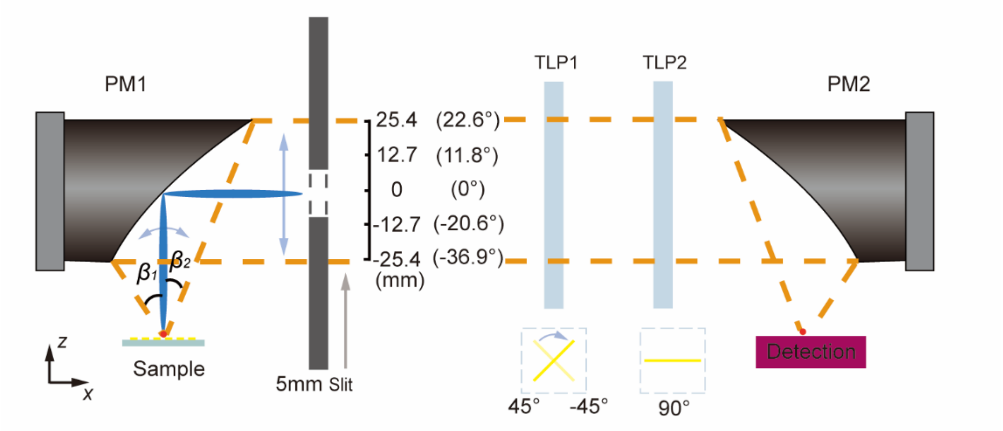

Fig. S5 Schematic of the core section of the experimental setup in Fig. S3 for measuring the angle-resolved terahertz (THz) intensity distribution. PM: parabolic mirror; TLP: THz linear polarizer.

Based on the above principle, the applied detailed measuring procedures are:

1) Set TLP1 to –45°;
2) Scan the slit in a 2-mm step along the z direction, and record the corresponding THz time-domain signals, i.e., $E_{-45°}(t, z)$;
3) Rotate TLP1 to 45°;
4) Repeat the procedure in step 2) and record $E_{45°}(t, z)$;
5) Convert the above measured THz time-domain signals to frequency-domain via Fast Fourier Transform (FFT) and extract the field information at the desired frequency f, i.e., $E_{-45°}(f, z)$ and $E_{45°}(f, z)$.
6) Calculate the circularly polarized THz field components by

$$\begin{bmatrix} E_l(f,z) \\ E_r(f,z) \end{bmatrix} = \frac{1}{\sqrt{2}} \begin{bmatrix} 1 & i \\ 1 & -i \end{bmatrix} \begin{bmatrix} E_{+45^\circ}(f,z) \\ E_{-45^\circ}(f,z) \end{bmatrix}. \quad \text{(S10)}$$

7) Calculate the final angle-resolved intensity distribution by squaring the field amplitudes ($|E_l|^2$ and $|E_r|^2$) and converting position z to emission angle α using Eq. (S9).

Note 5. Angular Resolution

The angular resolution reflects the system's ability to distinguish between two closely spaced angular directions, which is primarily determined by the far-field beamwidth. Larger resolution corresponds to smaller beam width. For our 1D demonstration, the beamwidth depends on the effective aperture length of the array, which is given by $L = N_x \cdot P_2$, where N_x is the number of sub-elements (SRR array) along the x-direction, and P_2 = 50 μm is the period of sub-element. The theoretical far-field beamwidth can be expressed as[S5]:

$$\Omega_{theo.} = \frac{\lambda}{L \cdot \cos(\alpha)} = \frac{\lambda}{N_x \cdot P_2 \cdot \cos(\alpha)} . \tag{S11}$$

where λ is the central wavelength and α is the beam deflection angle. To more intuitively illustrate the influence of array length on beamwidth, we perform beamwidth calculations for arrays with aperture lengths L of 2 mm, 4 mm, and 8 mm (corresponding to N_x = 40, 80, 160) at λ = 300 μm (1 THz) as an example, see Fig. S6a. It is seen that the beamwidth increases as the absolute deflection angle increases, while decreases as the aperture length increases.

In this work, the sample has a total length of 2 mm, corresponds to the black curve in Fig. S6a. The green squares and yellow triangles represent case 1 and case 2 in the main text, respectively. The corresponding theoretical beamwidths are $\Omega_{\text{theo.}}$ = 8.7° and 9.3°. To more clearly compare how the aperture length affects beamwidth, we further calculated the far-field radiation patterns using Eq. (2), as shown in Figs. S6b and S6c. The resulting beamwidths Ω_{cal} for Case 1 and Case 2 are observed to decrease from 7.8° and 8.3° (at L = 2 mm) to 3.9° and 4.1° (at L = 4 mm), and finally to 1.9° and 2.0° (at L = 8 mm). These results, represented by the red squares and blue triangles, are consistent with the theoretical prediction, see Fig. S6a.

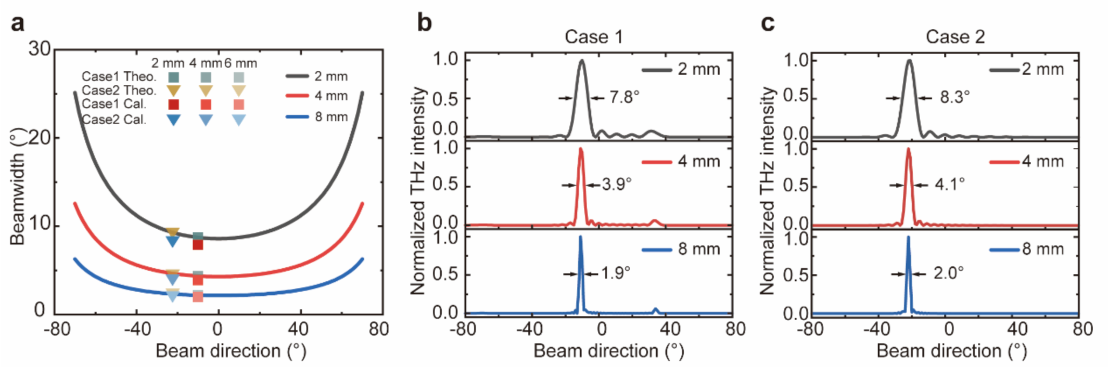

Fig. S6. Calculated beamwidths for photonic terahertz phased arrays (PTPAs) with different aperture lengths of 2 mm, 4 mm, and 8 mm. a Theoretical beamwidth at various emission angles. **b**,**c** Calculated far-field radiation patterns using the phase control schemes of case 1 and case 2 in the main text at 1.0 THz.

Note 6. Steerable range

The steerable range refers to the maximum angular span over which a phased array can effectively direct its main radiation beam while maintaining beam quality. This range is fundamentally constrained by the sub-element spacing and the phase gradient across the PTPA here. In our case, the sub-element spacing is P_2 = 50 μm, which satisfies $P_2 < \lambda/2$ for the central wavelength λ = 300 μm. This criterion effectively suppresses the formation of grating lobes within the designed steering range.

Considering the case of single beam steering, the maximum steerable range of a beam with wavelength λ is determined by the achievable Λ, according to the generalized Snell's law, i.e., $\alpha = \arcsin(\lambda/\Lambda)$[S6]. The beam steering here is implemented using four discrete sub-element phase states. To obtain a relative uniform linear phase gradient for efficient beam steering, the span of each phase state should better be the same, this constraint results in a phase period $\Lambda = 4mP_2$ with m = 1, 2, 3…

For a wavelength of λ = 300 μm, the minimum $\Lambda_{min} = 8P_2$ = 400 μm, corresponding to a maximum steerable range of −48.6° ~ 48.6°. Figure S7a illustrates the calculated far-field radiation patterns of different Λ using Eq. (2), where the angular-dependent gain is taken into account. It should be mentioned that larger steerable range is in principle achievable, if we ignore all the above constrains and apply non-uniform linear phase distribution, see Fig. S7b. The steerable range is enlarged to −59° ~ 59°. The corresponding DMD coding scheme for different phase states are schematically illustrated in Fig. S7c. The sub-element size P_2 is 50 μm. By selectively exciting the nonlinear PB sub-elements of desired orientations (denoted by different colors), THz waves with the corresponding phase can be generated. Based on this coding scheme, different phase periods Λ with uniform and non-uniform phase profiles are designed, as shown in Figs. S7d and S7e, respectively. Taking LCP THz waves as an example, such phase distributions enable beam steering toward the negative direction. By simply mirroring these spatial arrangements along the y direction, beam steering toward the positive direction can be achieved. When the phase distribution remains constant, as shown in Fig. S7f, the beam radiates towards the normal direction.

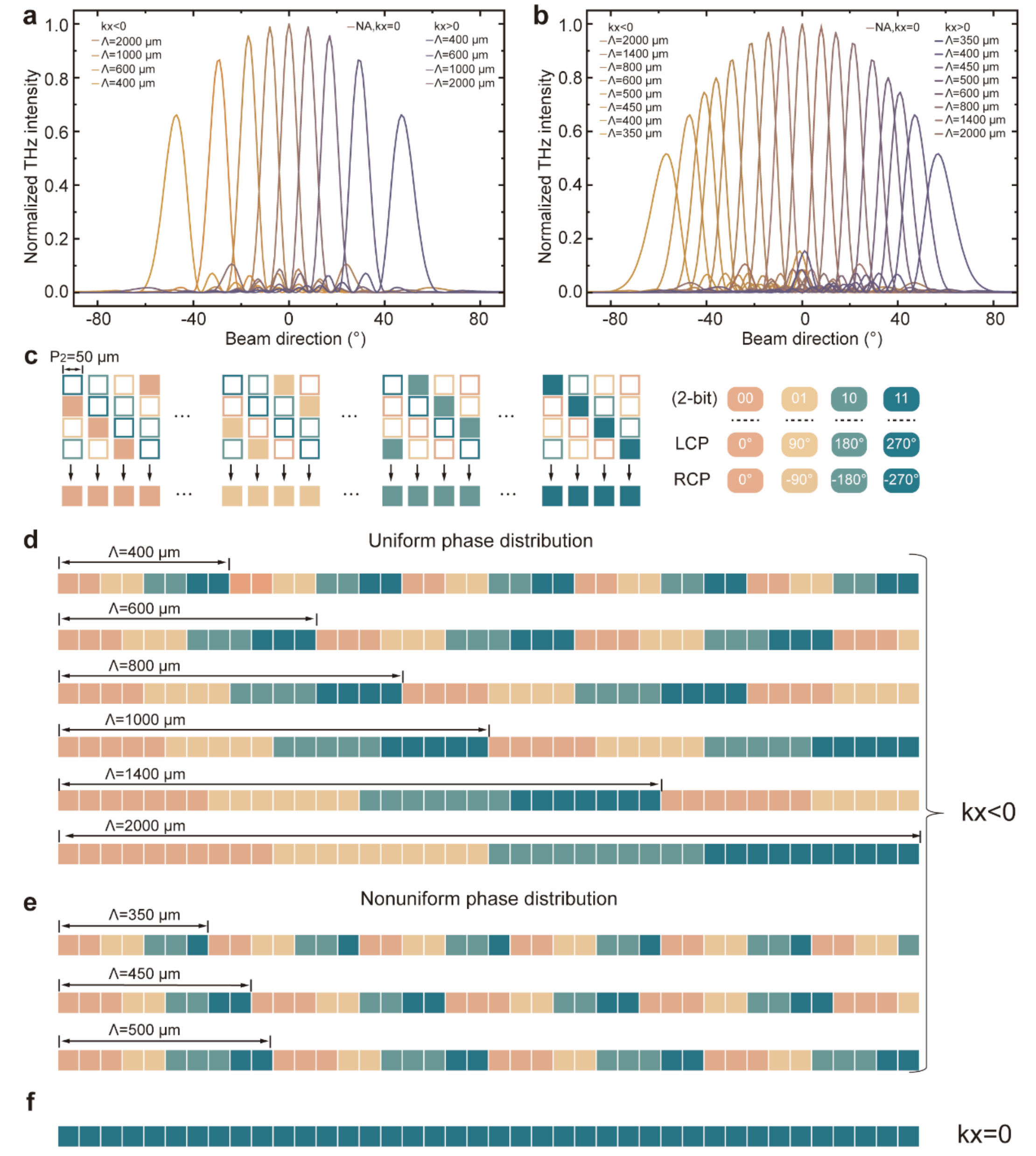

Fig. S7 Beam steerable range of the photonic terahertz phased array (PTPA). Calculated far-field radiation patterns of the PTPA using different phase period Λ with uniform **a** and non-uniform **b** phase distributions, respectively. **c** Schematic of the Digital Micromirror Device (DMD) coding scheme for different phase states, where the solid and hollow squares represent excited and non-excited nonlinear Pancharatnam–Berry (PB) sub-elements, respectively. **d**,**e** Equivalent uniform and nonuniform phase distributions of different phase periods Λ for calculating corresponding results of *kx* < 0 in **a** and **b**, respectively. **f** Equivalent constant phase distribution of $k_x = 0$.

Notice that, the beam steering angle cannot be continuously tuned, owing to the discrete feature of the phase control scheme. However, the angle step can be potentially reduced using smaller sub-element size P_2, which can be simply achieved here since the SRR size is only $P_1 = 382$ nm. This can also increase the steerable range based on the above control manner of the phase period, as smaller Λ can be achieved.

In summary, the beam steerable range is determined by the achievable phase period Λ, which can be improved using smaller sub-element size P_2. Other optimization methods may include applying certain designing algorithms of the phase distributions[S7]. Though the above analyses are carried out by taking 1.0 THz as an example, the same conclusion can be extended to the other THz frequencies.

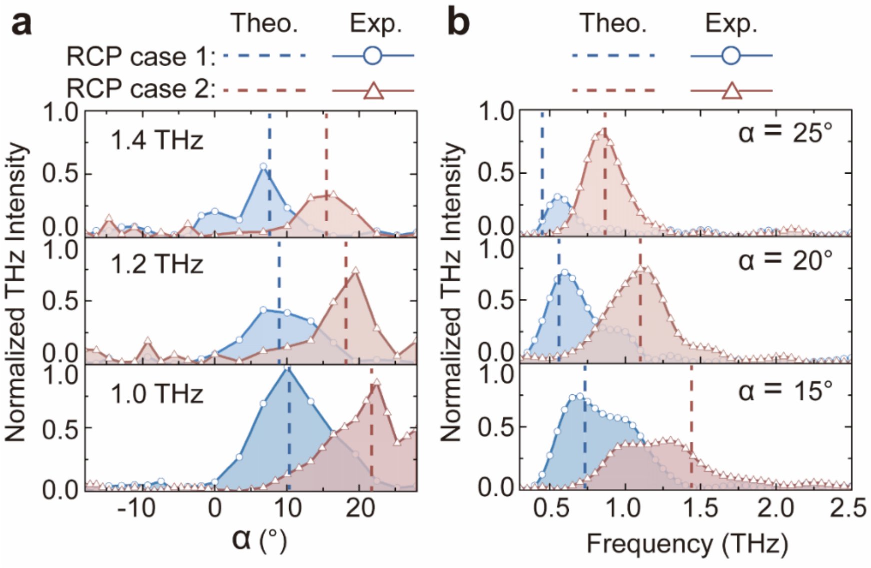

Fig. S8 Measured right-handed circularly polarized (RCP) terahertz (THz) single beamforming results for case 1 and case 2. a Measured intensity distributions of the generated RCP THz waves at 1.0, 1.2 and 1.4 THz as a function of deflection angle for case 1 and case 2, respectively. **b** Measured intensity spectra of the generated RCP THz waves at 25°, 20° and 15° for case 1 and case 2, respectively.

No.	Sign of k_x	Λ (μm)	Deflection angle (°)	Position on slit plane (mm)
1	< 0	150	–29.8	–19.4
2	< 0	200	–21.8	–13.5
3	< 0	250	–17.1	–10.4
4	< 0	300	–14.3	–8.6
5	< 0	350	–12.1	–7.3
6	< 0	400	–10.2	–6.1
7	< 0	450	–9.4	–5.6
8	< 0	500	–8	–4.8
9	< 0	600	–6.8	–4.0
10	< 0	700	–5.6	–3.3
11	< 0	800	–5.1	–3.0
12	< 0	900	–4.3	–2.5
13	< 0	1000	–3.1	–1.8
14	< 0	1500	–2.3	–1.4
15	= 0	NA	0	0
16	> 0	1500	2.3	2.5
17	> 0	1000	3.1	3.3
18	> 0	900	4.3	4.6
19	> 0	800	5.1	5.5
20	> 0	700	5.6	6.0
21	> 0	600	6.8	7.3
22	> 0	500	8	8.6
23	>0	450	9.4	10.1
24	>0	400	10.2	11.0
25	>0	350	12.1	13.1
26	>0	300	14.3	15.6
27	>0	250	17.1	18.8
28	>0	200	21.8	24.4
29	>0	150	29.8	35.0

Tab. S1 The 29 phase gradients applied to the slit imaging, whose values are indicated by the sign of k_x and Λ. The deflection angle and position on slit plane at 1.0 THz are determined by the phase gradients of various metasurface patterns, as calculated using Equation (1).

Note 7. Origins of the non-axial symmetric vortex intensity distributions

1) Influence from the discrete phases induced by selective excitation.

In an ideal case of Gaussian beam incidence with a 0.5-mm spot diameter (denoted as Gauss spot 1) for the pump and continuous helical phase distribution for the PTPA, see Fig. S9a, typical donut-shaped vortex intensity and helical phase distributions with good axial symmetry are observed, see Fig. S9b. The intensity distribution can be further homogenized by increasing the sample area. When discretizing the continuous phases into four levels (0°, 90°, 180°, 270°), see Fig. S9c, the intensity distribution degrades into octagon-shaped while the single phase singularity is divided into two, see Fig. S9d. Even so, the overall distributions still show relatively good axial symmetry.

However, upon further considering the selective excitation pattern to the pump spot by the DMD (see Fig. S9e), the intensity and phase distributions resemble the non-axial symmetric feature (see Fig. S9f). This can be attributed to the uneven sampling of the pump beam in different azimuthal angle ranges, which introduces discrete initial phase distribution.

2) Influence from the finite size of the PTPA sample.

The above uneven sampling is more obvious around the PTPA center according to our design. This can be improved by increasing the effective working area. As shown in Fig. S9g, when the working area is expanded to 2 mm × 2 mm and the Gaussian beam spot is enlarged to 1 mm in diameter (denoted as Gauss spot 2), a larger number of SRRs are excited within the illuminated region. The corresponding intensity and phase distributions show a vortex beam with much better quality in axial symmetry, see Fig. S9h. This can be improved by further increasing the working area through increasing the sizes of the Gaussian spot and the PTPA.

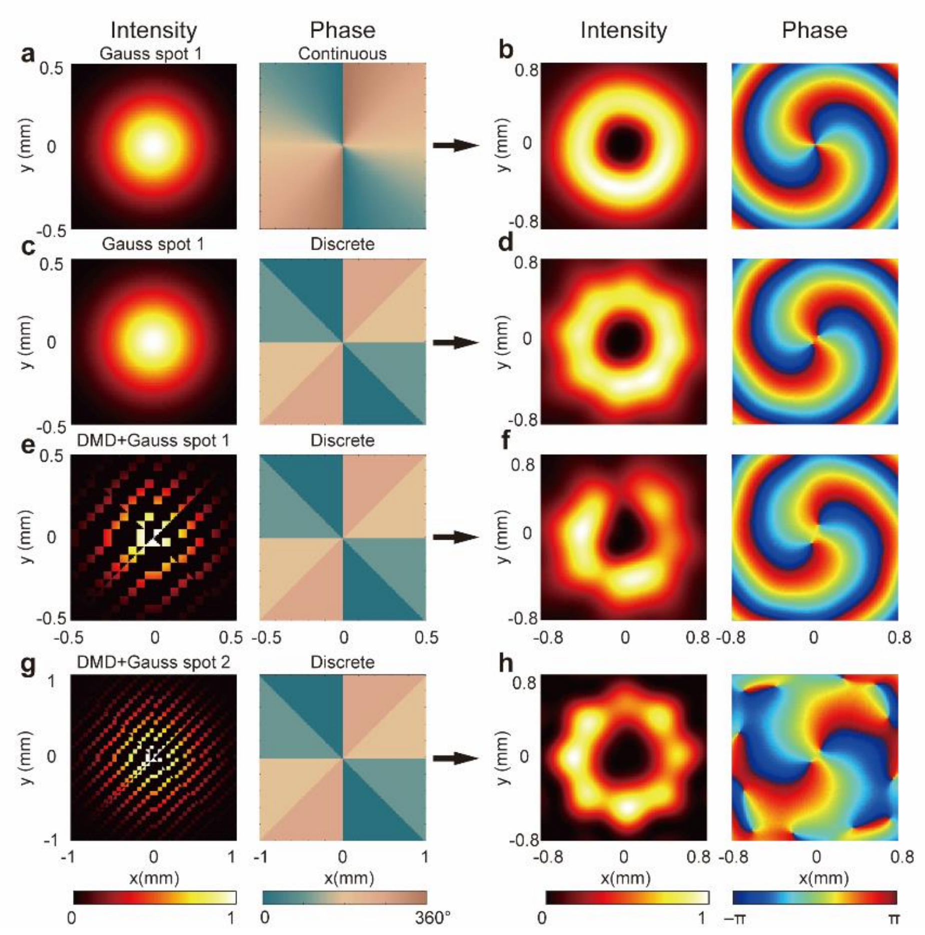

Fig. S9 Calculated intensity and phase distributions of the generated terahertz (THz) vortex beams carrying a topological charge of $l = +2$ at 1.0 THz under various excitation conditions. **a**,**c**,**e**,**g** Excitation conditions of Gaussian spot 1 (0.5-mm spot diameter) with a continuous helical phase distribution **a**, Gaussian spot 1 with a discretized phase distribution **c**, Gaussian spot 1 with a discretized phase distribution considering the selective excitation of the Digital Micromirror Device (DMD) **e**, and Gaussian spot 2 (1.0-mm spot diameter) with a discretized phase distribution **g**, respectively. All the discretized phase distributions are composed of four-level phase of 0°, 90°, 180°, and 270°. **b**,**d**,**f**,**h** Calculated intensity and phase distributions under the excitation conditions in **a**,**c**,**e**,**g**, respectively. All the results are obtained using Rayleigh–Sommerfeld diffraction theory at a propagation distance of 0.7 mm.

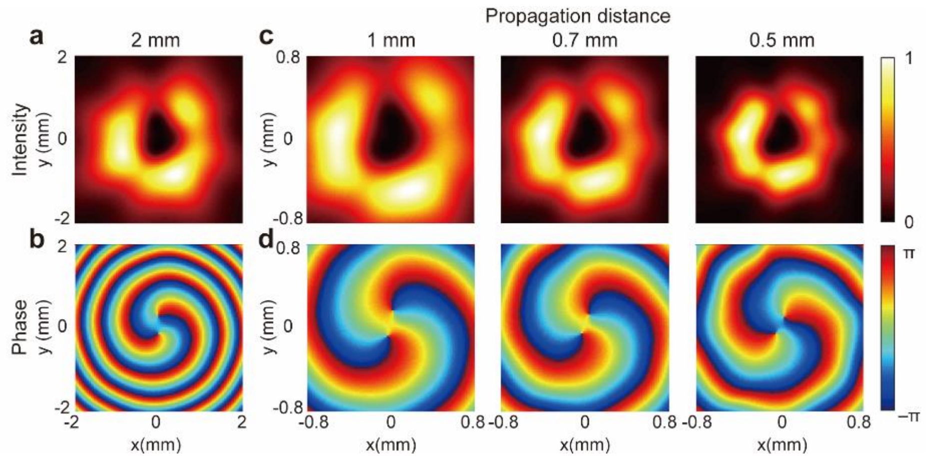

Fig S10. Calculated intensity and phase distributions of the generated terahertz (THz) vortex beams carrying a topological charge of *l* = +2 at 1.0 THz at different propagation distances. a,**b** The intensity and phase distributions at a propagation distance of 2 mm with an imaging area of 4 mm × 4 mm. **c**,**d** The intensity and phase distributions at propagation distances of 1 mm, 0.7 mm, and 0.5 mm, respectively, with an imaging area of 1.6 mm × 1.6 mm. It is seen that the calculated results at 0.7 mm distance are more consistent with the corresponding measured results in Fig. 6h, indicating that the measured plane should be closer to the image plane of the fields about 0.7 mm away from the photonic THz phased array (PTPA) sample with respect to the 4*f* system.

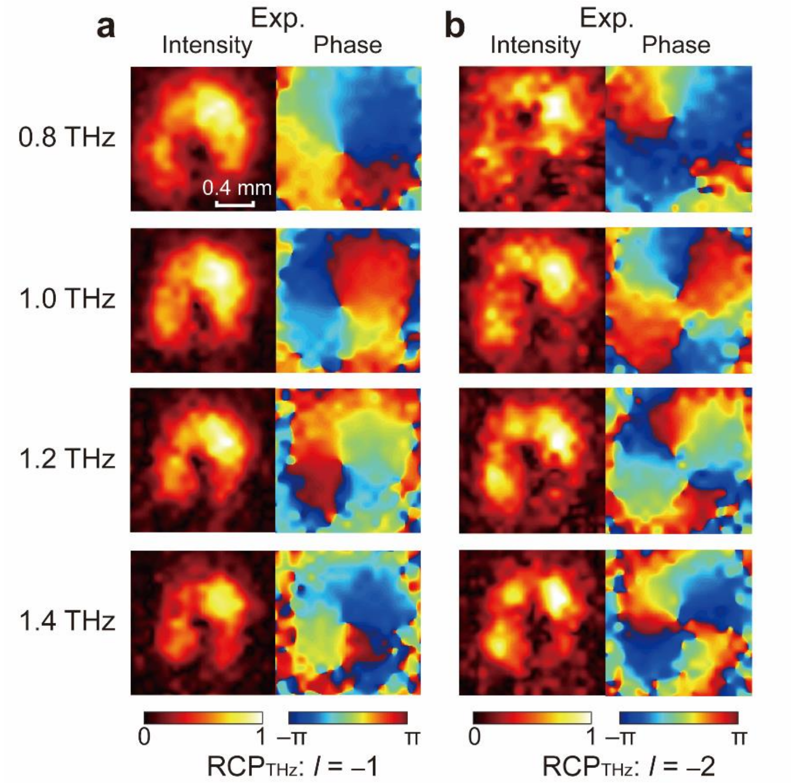

Fig. S11 Measured right-handed circularly polarized (RCP) terahertz (THz) vortex beam generation. a,b Measured transverse intensity and phase distributions of the generated RCP THz beams at 0.8, 1.0, 1.2, and 1.4 THz for case 6 and case 7, respectively. The scalebar in the top-left panel of Fig. S11 is applicable to all the results.

Note 8 Efficiency of the nonlinear metasurface.

Due to the lack of conditions for directly measuring the power of the generated THz wave, the efficiency of the nonlinear metasurface was evaluated indirectly by comparing its THz emission with that of a conventional ZnTe crystal[S8]. Without loss of generality, the nonlinear metasurface sample is a uniform SRR array with the same orientation, while the ZnTe crystal is <110>-cut with a thickness of 200 μm.

Figure S12a shows the corresponding measured THz peak-to-peak amplitudes as a function of pump wavelength at 1275 nm, where the result of the ZnTe crystal under 800 nm pump is also presented. It is seen that the responses of the ZnTe crystal under 1275 nm and 800 nm pumps are nearly the same, while the response of the nonlinear metasurface exhibits a comparable level, although a saturation effect emerges at higher fluences. This observation aligns with previously reported results[S2,S9,S10].

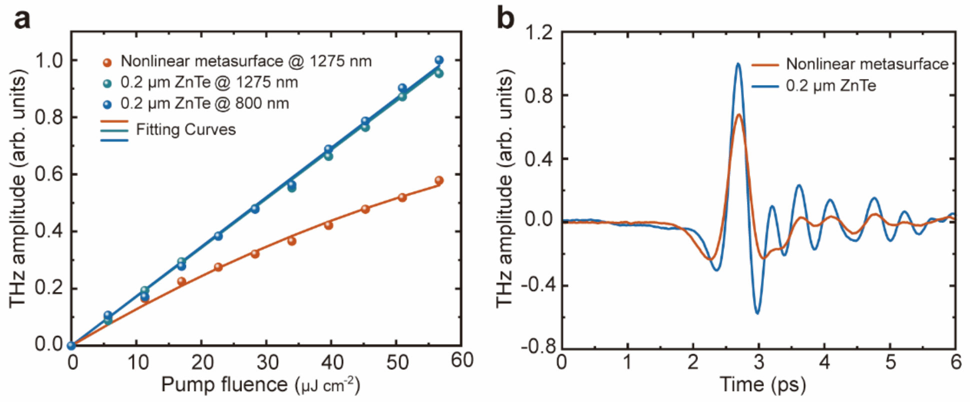

Fig. S12 Measured terahertz (THz) generations from the proposed nonlinear metasurface and ZnTe crystal. a Measured pump-fluence-dependent THz peak-to-peak amplitudes of the nonlinear metasurface sample under 1275 nm pump, and a 200 μm-thick ZnTe crystal under 800 nm and 1275 nm pump. **b** Measured time-domain signals of the sample and ZnTe crystal (at 800 nm) at 56.6 μJ cm^{-2} pump fluence.

According to Ref. [S8], a 500 μm-thick ZnTe crystal achieves a conversion efficiency of 3×10^{-5} under a 1.4 mJ/cm^2 pump at 800 nm. In our case, the THz peak-to-peak amplitude of the nonlinear metasurface is approximately 0.58 of that of the ZnTe under a 56.6 μJ/cm^{-2} pump, as shown in Fig. S12b. Taking into account the

differences in pump fluence, sample thickness, and relative THz field amplitude, the efficiency is roughly estimated to be $3 \times 10^{-5}/(25 \times 2.5^2 \times 1.72^2) = 6.5 \times 10^{-8}$, where the factors of 25, 2.5, and 1.72 correspond to the pump fluence ratio, thickness ratio of ZnTe in Ref.[S8] and here, and THz field amplitude ratio of ZnTe and nonlinear metasurface in Fig. S12b, respectively. Although the absolute efficiency is modest, it is critical to emphasize that our nonlinear metasurface is only 48 nm thick, which is four-orders thinner than the ZnTe crystal. This implies a remarkably high effective nonlinear response. In specific, the effective second-order susceptibility of the nonlinear metasurface can be ~541 times of ZnTe.

It is clear that higher efficiency gives rise to stronger THz radiation, which in turn improves the dynamic range and signal-to-noise ratio of the PTPA, and thus the beam steering performance. Regarding the potential trade-offs between performance and efficiency of our nonlinear metasurface: the first one should be the saturation effect, which means that the efficiency cannot keep a large increasing trend as the pump fluence; the second one should be the low damaging threshold feature of nonlinear metasurfaces based on plasmonic structures, where excessive pump fluence will bring irreparable damage to the sample.

Reference

S1. Boyd, R. W. *Nonlinear Optics* (Academic Press, 2020).

S2. Lu, Y. et al. Integrated Terahertz Generator-Manipulators Using Epsilon-near-Zero-Hybrid Nonlinear Metasurfaces. *Nano Lett.* **21**, 7699-7707 (2021).

S3. Jia, W. et al. Polarization-entangled Bell state generation from an epsilon-near-zero metasurface. *Sci. Adv.***11**, eads3576 (2025).

S4. Deng, Z. et al. Full-Color Complex-Amplitude Vectorial Holograms Based on Multi-Freedom Metasurfaces. *Adv. Funct. Mater.* **30**, 1910610 (2020).

S5. Mailloux, R. *Phased Array Antenna Handbook* (Arterch House, 2017).

S6. Yu, N. et al. Light propagation with phase discontinuities: generalized laws of reflection and refraction. *Science* **334**, 333-337 (2011).

S7. Wen, Y., Wang, B. & Ding, X. A Wide-Angle Scanning and Low Sidelobe Level Microstrip Phased Array Based on Genetic Algorithm Optimization. *IEEE Trans. Antennas Propag*. **64**, 805-810 (2016).

S8. Blanchard, F. et al. Generation of 1.5 μJ single-cycle terahertz pulses by optical rectification from a large aperture ZnTe crystal. *Opt. Express* **15**, 13212-13220 (2007).

S9. Wang, Q. et al. Nonlinear Terahertz Generation: Chiral and Achiral Meta-Atom Coupling. *Adv. Funct. Mater.* **33**, 2300639 (2023).

S10. McDonnell, C., Deng J., Sideris S., Ellenbogen T. & Li, G.. Functional THz emitters based on Pancharatnam-Berry phase nonlinear metasurfaces. *Nat. Commun.* **12**, 30 (2021).
